# Biographical Sketch of the Late Prof Robert Hare, M. D.

**Published:** 1858-10

**Authors:** 


### *Biographical Sketch of the late* Prof. ROBERT HARE, M. D.

The grave has hardly closed over the remains of one talented and eminent member of the faculty, ere we are called to mourn for another, one equally well known to science, and whose fame has extended throughout the civilized world.

Dr. Hare's life has been one of unremitting labor in the cause of science, and by his ability and research he has contributed a large number of valuable discoveries to the list of the present century.

He was born in Philadelphia, in 1781. His father was an extensive brewer, and young Hare, for some time, was engaged in this occupation also. He entered the Chemical Department of the Pennsylvania University about the year 1800, and that he here well employed his time is evinced by the fact that only two years after, he contributed his first invention, the oxyhydrogen blow-pipe, an apparatus by which he was enabled to reduce the hardest substances, and thus evolved many of the metallic bases. He communicated to the Chemical Section of the British Association for the Advancement of Science, while on a visit to England, the fact of his having thus reduced 25 ounces of platina to a fluid state.

For this instrument he was awarded, by the American Academy of Arts and Sciences, the Rumford medal. He subsequently modified it so as to feed it with alcohol.

A very important use of this instrument was afterwards discovered by Lieut. Drummond, who, by the introduction of a piece of lime into the flame thus produced, was enabled to give to science the celebrated "lime light," better known as the "Drummond light,"

About the year 1819, he produced a new and valuable galvanic instrument, well known as "Hare's calorometer," concerning which, he published a detailed account in *Silliman's Journal.* A year or two later, by the same medium, he announced his new galvanic theory, together with a description of a new galvanic instrument, to which he gave the name of the "Galvanic Deflagrator," commonly called "Hare's Deflagrator." By this also he fused platina, etc.

In a little while we find him contributing a new and much improved gasometer, a eudiometer, a litrometer, to ascertain the specific gravity of various fluids, the hydrostatic blowpipe, the single gold-leaf electroscope, and a host of smaller instruments, or improvements in old instruments.

Nor was it only to chemistry that he made such valuable additions, for in materia medica we are indebted to him for many important improvements, etc. Thus, we have the process for denarcotizing laudanum, and a method of detecting very minute quantities of opium while in solution.

In 1818, he succeeded Dr. John Redman Coxe in the chair of chemistry in the Medical Department of the Pennsylvania University, which he filled with an increasing reputationtill 1847, a period of twenty-nine years. As he never published any systematic work (his *Compendum of Chemistry* being only a text-book for his pupils,) these lectures are the only record of his many and brilliant discoveries, but they alone are sufficient among the immense number of pupils who have profited by his instruction, to cause him to live forever as a model of industrous and untiring labor in the cause of science.

After his resignation from the University, at which he was elected Emeritus Professor of Chemistry in that school, he paid much attention to meteorology, and read several papers before the American Philosophical Association on tornadoes, in which he attributed the atmospheric disturbances to an electrified current of air. As all new theories meet with more or less violent opposition, of course this was not destined to be received quietly, and on its promulgation, he encountered a storm almost as great as one of the tornadoes for which he was endeavoring to account; and many fierce conflicts occurred at the meetings of the society, between Dr. Hare and his rival investigators.

For more than fifty years he has been an active scientific man, though these pursuits did not prevent him from finding time to mingle in society, and enjoy the relaxation of domestic pleasures. By his talented mind, he rendered himself agreeable in conversation, and being of a prepossessing manner, he made for himself hosts of friends.

If, in the weakness of age, he may have fallen into any of the popular errors, we must recall the past, and reflect upon the numberless services he has rendered the world, while yet in his prime, and we will thus find sufficient to counterbalance all his error.

He died in his native city, on Saturday, May 15th, 1858, in his seventy-seventh year, of typhoid pneumonia.—*Med. & Sur. Reporter*

